# Sex hormone-binding globulin promotes the osteogenic differentiation potential of equine adipose-derived stromal cells by activating the BMP signaling pathway

**DOI:** 10.3389/fendo.2024.1424873

**Published:** 2024-10-17

**Authors:** Jennifer M. Irwin-Huston, Lynda Bourebaba, Nabila Bourebaba, Artur Tomal, Krzysztof Marycz

**Affiliations:** ^1^ Department of Experimental Biology, Faculty of Biology and Animal Science, Wrocław University of Environmental and Life Sciences, Wrocław, Poland; ^2^ International Institute of Translational Medicine, Wisznia Mała, Poland; ^3^ Department of Medicine and Epidemiology, School of Veterinary Medicine, University of California, Davis, Davis, CA, United States

**Keywords:** osteogenesis, ASCs, SHBG, BMP, mitochondrial dynamics, autophagy

## Abstract

**Background:**

Musculoskeletal injuries and chronic degenerative diseases pose significant challenges in equine health, impacting performance and overall well-being. Sex Hormone-Binding Globulin (SHBG) is a glycoprotein determining the bioavailability of sex hormones in the bloodstream, and exerting critical metabolic functions, thus impacting the homeostasis of many tissues including the bone.

**Methods:**

In this study, we investigated the potential role of SHBG in promoting osteogenesis and its underlying mechanisms in a model of equine adipose-derived stromal cells (ASCs). An SHBG-knocked down model has been established using predesigned siRNA, and cells subjected to osteogenic induction medium in the presence of exogenous SHBG protein. Changes in differentiation events where then screened using various analytical methods.

**Results:**

We demonstrated that SHBG treatment enhances the expression of key osteoconductive regulators in equine ASCs CD34^+^ cells, suggesting its therapeutic potential for bone regeneration. Specifically, SHBG increased the cellular expression of BMP2/4, osteocalcin (OCL), alkaline phosphatase (ALP), and osteopontin (OPN), crucial factors in early osteogenesis. Furthermore, SHBG treatment maintained adequate apoptosis and enhanced autophagy during osteogenic differentiation, contributing to bone formation and remodeling. SHBG further targeted mitochondrial dynamics, and promoted the reorganization of the mitochondrial network, as well as the expression of dynamics mediators including PINK, PARKIN and MFN1, suggesting its role in adapting cells to the osteogenic milieu, with implications for osteoblast maturation and differentiation.

**Conclusion:**

Overall, our findings provide novel insights into SHBG’s role in bone formation and suggest its potential therapeutic utility for bone regeneration in equine medicine.

## 1 Introduction

Sex hormone-binding globulin (SHBG) is a glycoprotein that plays a crucial role in regulating the bioavailability of sex steroids within the circulatory system. It is synthesized primarily in the liver and has a high affinity for binding androgens and estrogens, thus controlling the balance of sex hormones in the body. Structurally, SHBG is composed of two identical subunits that form a homodimer, and each subunit consists of approximately 373 amino acids. The protein has a carbohydrate moiety attached, essential for its proper function and stability (1). Its molecular role is multifaceted: it acts as a transport protein that carries sex hormones in the bloodstream, preventing premature metabolism and excretion and facilitating their delivery to target tissues (2). Additionally, SHBG modulates the hormonal signal by controlling the amount of free and active hormones that can pass into cells. The structure of SHBG includes a steroid-binding domain, which allows for a reversible interaction with hormones, thereby influencing their activity and the physiological responses they regulate, such as reproduction, development, and metabolism (3).

Through its high-affinity binding to androgens and estrogens, SHBG regulates the fraction of circulating sex hormones in a bioactive, unbound state, capable of engaging with osteoblastic and osteoclastic receptors. The consequent hormonal action on these effector cells underlines critical processes of bone remodeling and maintenance of skeletal integrity. At a molecular level, SHBG influences osteogenic activity by dictating the equilibrium between free and bound hormonal forms, thereby controlling the extent of interaction with specific bone cell receptors (4, 5). Emerging evidence posits a more direct role for SHBG in bone physiology beyond its canonical function of steroids transport. The presence of SHBG receptors on osteoblasts indicates a direct signaling mechanism whereby SHBG, independent of its hormone cargo, may initiate intracellular cascades that influence bone formation and resorption. Such a mechanism could involve the modulation of gene expression profiles pertinent to osteogenesis and bone mineral homeostasis. The bioavailable hormones engage intracellular signaling pathways, such as the bone morphogenetic protein (BMP) axis, a pivotal determinant of osteoblast differentiation and maturation, integral to bone anabolism (4, 6).

BMP family, especially BMP-2, BMP-4, and BMP-7, plays an essential role in bone formation, remodeling, and repair, exerting multifaceted influences on osteoblastic activity (7). BMP-2 is the most extensively characterized, known to induce the commitment of pluripotent mesenchymal cells to the osteoblast lineage and promote their differentiation and maturation. It stimulates cellular responses that culminate in the synthesis of bone matrix proteins and enhances mineral deposition (8). Clinical applications of BMP-2 have included its use in spinal fusions and the treatment of non-union fractures, where its potent osteogenic capability supports the healing process (9). Like BMP-2, BMP-4 contributes to the early stages of osteoblast differentiation but also plays a distinct role in limb patterning and chondrogenesis during embryonic development. Its expression levels and gradient patterns are crucial for the adequate formation of skeletal structures (8). In the context of bone homeostasis, BMP-4’s influence extends to the regulation of apoptosis in osteoblasts, thus affecting the formation and survival of these cells. BMP-7, also termed osteogenic protein-1 (OP-1), is another critical player in skeletal development and regeneration. Its osteogenic activity aids in repairing bone defects and is pivotal in the recovery of skeletal integrity following injury (10, 11). BMP-7 has been shown to reverse the effects of bone loss in osteoporotic models, indicating its potential therapeutic utility. Moreover, its ability to enhance the re-establishment of bone vasculature denotes a significant role in coupling angiogenesis with osteogenesis, further underscoring its integral involvement in bone health and disease mitigation (12).

Adipose-derived mesenchymal stem cells (ASCs) are a population of stromal stem cells capable of differentiating into various cell lineages, including osteoblasts, which positions them as a promising resource for bone regenerative therapies (13). These cells are traditionally characterized by the high expression of specific surface markers such as CD29, CD44, CD90, and CD105 while lacking the hematopoietic markers CD14, CD34, and CD45, delineating their unique molecular identity. However, recent research revealed that freshly isolated ASCs can transiently express the CD34 surface protein, particularly in their early stages of isolation, indicating a distinct subpopulation postulated to exhibit higher proliferation and differentiation capacities, thereby contributing to their unique regenerative potential (14).

ASCs are particularly appealing for clinical applications due to their accessibility, abundance in adipose tissue, and potent proliferative capacity (12, 15, 16). Their role in bone regeneration is mediated through the secretion of growth factors and cytokines that facilitate the recruitment, proliferation, and differentiation of osteoprogenitor cells and the formation of new blood vessels, a process essential for nourishing and surviving newly formed bone tissue (17).

In the molecular orchestration of bone repair, ASCs interact with BMP signaling pathways, a critical determinant of osteoblast differentiation. They also secrete extracellular matrix proteins and support the mineralization process necessary for bone matrix development. The osteogenic potential of ASCs can be enhanced by genetic modification or specific growth factors, which upregulate osteogenic genes and promote the osteoblastic phenotype (18, 19). Furthermore, the immunomodulatory properties of ASCs contribute to a conducive regenerative environment by modulating the local immune responses, reducing inflammation, and thus fostering an optimal setting for bone healing and regeneration (20).

The application of various proteins to induce and improve the osteogenic differentiation potential of ASCs is a focal aspect of regenerative medicine. Proteins such as autophagy regulators, including rapamycin and bafilomycin, have been investigated for their roles in modulating cellular processes like autophagy and mitophagy, which are crucial for maintaining cellular homeostasis and enhancing differentiation capabilities (21). Autophagy activation is associated with removing dysfunctional organelles and proteins, thus promoting a conducive cellular environment for osteogenic differentiation. Similarly, the induction of mitophagy, the selective autophagy of mitochondria, is vital for the metabolic reprogramming of ASCs towards osteoblastic lineage commitment and maturation (22).

The present study aimed to explore the role of SHBG on the osteogenic differentiation potential of ASCs cellular pathways like autophagy and mitophagy, aiming to reveal new insights into the regulation of osteogenesis. This research may identify potential therapeutic targets for bone regeneration and the treatment of musculoskeletal degenerative diseases.

## 2 Materials and methods

### 2.1 Isolation of equine ASCs CD34^+^ cells

Equine ASCs were isolated from the subcutaneous adipose tissue of healthy adult horses using collagenase type I (Gibco, Warsaw, Poland). First, tissue fragments were washed in phosphate buffered saline (PBS) (Sigma-Aldrich, Poznan, Poland) containing 1% penicillin-streptomycin (P/S) solution (Gibco, Warsaw, Poland). Then, the tissue fragments were mechanically minced and incubated in a digestion solution containing DMEM-F12 w/L-glutamine w/15 mM HEPES (Biowest, Gdańsk, Poland) and 1 mg/ml of type I collagenase (Gibco, Warsaw, Poland). The suspension was then incubated at 37°C for 40 minutes; after that, the mixture has been filtered through a 70 µm cell strainer and centrifuged at 1500g for 10 minutes. The cell pellet was washed with PBS and centrifuged again at 300 for 4 minutes. The resulting total ASCs were then subjected to CD34 positive cell isolation using the Dynabeads CD34 Positive Isolation Kit (Invitrogen ™, Poland) according to the manufacturer’s instructions, immediately after isolation in order to selectively enrich the CD34+ subpopulation for subsequent experiments. Briefly, the cells were suspended in 3 ml isolation buffer, washed and incubated with the dynabeads at 4°C for 30 minutes with gentle tilting and rotation. Dynabead-bound cells (CD34^+^ cells) were then separated using an EasySep™ Magnet and the supernatant was discarded. Cells were washed twice in the buffer and then resuspended in 100 µl of DERACHaBEAD, the mixture has been incubated at room temperature for 45 minutes under rotation. Then, 2 ml of isolation buffer were added and mixed to the previous solution. Dynabeads were separated on an EasySep™ Magnet and the supernatant containing the CD34^+^ cells was collected and washed with isolation buffer. Cells were centrifuged at 400g for 10 minutes and resuspended in culture medium consisting of Dulbecco′s modified Eagle′s medium - low glucose (containing 1000 mg/L of glucose) (Sigma-Aldrich, Poznan, Poland) supplemented with 5% of fetal bovine serum (FBS) (Sigma-Aldrich, Poznan, Poland) and 1% of P/S. The cells were maintained under standard conditions (37°C, 5% CO_2_ and 95% humidity) and media were changed every 2 days.

### 2.2 Cell culture and osteogenic differentiation

Cells were seeded in either 24- or 6-well plates, and cultured under standard conditions to an approximate 80% confluence. Thereafter, SHBG protein expression in the cells was silenced using small interfering RNA (Silencer Select Pre-designed siRNA, Ambion, exon 5) at a concentration of 20 nM mixed with Lipofectamine RNAiMAX reagent (Invitrogen ™, Poland). SHBG gene expression was knocked down for 72 hours. The cells were then subjected to osteogenic differentiation using the StemPro Osteogenesis Differentiation Kit (Gibco, Warsaw, Poland). Differentiation was performed for 21 days, and differentiation medium changed every 4 days. Three experimental groups were included as follows: CTRL: ASCs untreated native cells; siSHBG_UT_: ASCs cells with silenced SHBG; siSHBG_T_: ASCs cells with silenced SHBG and treated with 50 nM exogenous SHBG protein (Fitzgerald, United Kingdom). Additionally, a mock-transfected control group (transfected with Lipofectamine RNAiMAX without siRNA) was investigated under similar conditions, which did not differ significantly from the untreated native cells (data not included).

Non-differentiating cells were incubated for 24 hours in test medium consisting of Dulbecco′s modified Eagle′s medium - low glucose supplemented with 0.2% Bovine Serum Albumin Solution (BSA) (Sigma-Aldrich, Poznan, Poland) and 1% P/S. SHBG protein at a concentration of 50 nM was also added to the siSHBG_T_ group.

### 2.3 Analysis of SHBG protein levels post-transfection

To verify the effectiveness of SHBG silencing in ASCs, transfected and untransfected cells were lysed in RIPA buffer containing protease and phosphatase inhibitors cocktail on ice, and centrifuged to remove cell debris. Protein concentrations were quantified from supernatants using the Pierce™ BCA Protein Assay Kit. For protein analysis, lysates were mixed with Laemmli buffer, denatured, and subjected to SDS-PAGE. Separated proteins were afterwards transferred to PVDF membranes and blocked with a 5% non-fat milk solution during 1h at room temperature. Detection of proteins was performed using SHBG and β-actin primary antibodies (Anti-SHBG, 1:1000, Biorbyt orb11366; anti- β-actin, 1:5000, Merck a5441) and HRP-conjugated secondary antibodies. Chemiluminescent signals were captured with the ChemiDoc MP Imaging System and analyzed using Image Lab Software.

### 2.4 Analysis of cell viability and proliferation

The viability of cultured undifferentiated cells was assessed using the resazurin-based assay kit (TOX8) (Sigma-Aldrich, Poznan, Poland) following manufacturer’s protocol. Briefly, the culture media were replaced with fresh culture medium containing 10% resazurin dye and cells were incubated for 2 hours at 37°C, 5% CO_2_ and 95% humidity. Absorbances were measured spectrophotometrically (Epoch, Biotek, Bad Friedrichshall, Germany) at a wavelength of 600 nm for resazurin and 690 nm reference wavelength.

Cell proliferation was analyzed using the BrdU Cell Proliferation ELISA Kit colorimetric assay (Abcam, Gdańsk, Poland) according to the manufacturer’s instructions. Absorbance was measured at two wavelengths, 450 nm and 550 nm, using spectrophotometer (Epoch, Biotek, Bad Friedrichshall, Germany).

### 2.5 Cytometric evaluation of oxidative stress

Oxidative stress analysis involved the quantification of reactive oxygen species (ROS) and nitric oxide (NO) accumulation. ROS accumulation was assessed using the Muse™ Oxidative Stress Kit (Luminex, Poznań, Poland) according to the manufacturer’s instructions. Briefly, for ROS, 10^6^ cells were mixed with the Muse™ Oxidative Stress Working Solution, followed by a 30 min incubation at 37°C. The Working Solution contains dihydroethidium (DHE), which segregates cells into two sub-populations: ROS- and ROS+. Regarding the NO accumulation, 10^7^ cells were incubated with the Muse^®^ Nitric Oxide working solution (Muse Nitric Oxide Reagent 1:1000 with 1x Assay Buffer) at 37°C during 30 min; then, the Muse^®^ 7-AAD working solution was added to the samples and analyzed using the Muse™ Cell Analyzer Merck, Germany).

### 2.6 Visualization of cells’ organelles

Cells were stained with MitoRed dye (Sigma-Aldrich, Poznan, Poland) at a ratio of 1:1000 in culture medium and incubated for 30 minutes at 37^°^C in the dark. Cells were then washed in PBS and fixed in 4% paraformaldehyde (PFA) during 40 minutes at room temperature in the dark. After that, cells were permeabilized in PBS with 0.1% Triton X-100 solution (Sigma-Aldrich, Poznan, Poland) for 15 minutes. Then cells were incubated with atto-488-labeled phalloidin (Sigma-Aldrich, Poznan, Poland) (1:800 in PBS) for 40 min at room temperature in the dark. Finally, the nuclei were counterstained with Fluoroshield with DAPI (Sigma-Aldrich, Poznan, Poland). Obtained samples were visualized and analyzed using the confocal microscopy (Observer Z1 Confocal Spinning Disc V.2 Zeiss), as well as the ImageJ Software. Furthermore, the mitochondria morphology analysis was conducted on the cells of each experimental group using the MicroP software (ver. 1.1.11b, Biomedical Image Informatics Lab, Taipei City, Taiwan (R.O.C.) Institute of Biomedical Informatics, National Yang Ming Chiao Tung University) powered by MATLAB (versionR2010b, The Math Works, Natick, MA, USA).

### 2.7 Ultrastructural analysis of differentiated cells

The specimens for ultrastructural analysis were initially fixed in 2.5% glutaraldehyde and subsequently rinsed in a 0.1 M phosphate buffer (pH = 7.2) after 21 days of osteogenic differentiation. Following dehydration in graded ethanol and drying in a laminar chamber, each sample was coated with gold using a SCANCOAT 6, EDWARDS (Crawley, England) sputter coater. The structural examination was conducted using a scanning electron microscope (SEM; Zeiss EVO LS15) and focused ion beam (FIB; Zeiss, Cobra, AURIGA 60).

### 2.8 Alizarin red staining

The cells were fixed in 4% PFA for 10 minutes at room temperature. At day 0 and day 21 of osteogenesis. After that, the cells were washed with distilled water and stained with 2% alizarin red solution. After 10 minutes, cells were washed several times with distilled water and photographed under an inverted light microscope (AxioObserverA1; Zeiss, Oberkochen, Germany).

For quantitative analysis, bound Alizarin Red dye was extracted from stained cells with a 10% acetic acid solution for 30 min at room temperature with gentle shaking to ensure complete extraction. After the incubation period, the dye extract was transferred to a microcentrifuge tube and neutralized with an equal volume of 10% ammonium hydroxide. Absorbances were afterwards measured at 405 nm using microplate spectrophotometer (Epoch, Biotek, Bad Friedrichshall, Germany) (23).

### 2.9 Immunofluorescence staining

Cells were fixed in 4% PFA during 40 minutes at room temperature in the dark at day 21 of osteogenesis. They were then washed three times with PBS and permeabilized in PBS supplemented with 0.1% Triton X-100 solution (Sigma-Aldrich, Poznan, Poland) for 15 minutes at room temperature. The cells were then washed again in PBS and incubated with primary antibodies (**Table 1**) overnight at 4^°^C. The cells were then washed twice with PBS and incubated with secondary antibodies (**Table 1**) during 1 hour at room temperature. Obtained samples were counterstained with Fluoroshield with DAPI (Sigma-Aldrich, Poznan, Poland), visualized with confocal microscopy (Observer Z1 Confocal Spinning Disc V.2 Zeiss), and processed with ImageJ Software.

**Table 1:** List of antibodies used for immunofluorescence staining.

Antibody	Concentration	Catalog No	Company
BMP-2/4 Antibody (H-1)	1:100	sc-137087	Santa Cruz
Anti-BMP7 antibody produced in rabbit	1:100	sab2107883	Sigma Aldrich
OPN Antibody (AKm2A1)	1:100	sc-21742	Santa Cruz
RUNX2 Antibody (M-70)	1:100	sc-10758	Santa Cruz
COL1A1 Antibody (3G3)	1:100	sc-293182	Santa Cruz
Goat Anti-Rabbit IgG H&L (Alexa Fluor^®^ 488)	1:800	ab150077	Abcam
Alexa Fluor^®^ 488 AffiniPure Rabbit Anti-Mouse IgG (H+L)	1:800	315-545-003	Jackson ImmunoResearch

BMP-2/4, Bone Morphogenetic Protein 2/4; BMP-7, Bone Morphogenetic Protein 7; OPN, Osteopontin; RUNX2, Runt-Related Transcription Factor 2; COL1A1, Collagen, Type I, Alpha.

### 2.10 Gene expression analysis

Total RNA was extracted from differentiated cells using the EXTRAzol^®^ reagent (Blirt DNA, Gdańsk, Poland) as per the manufacturer’s guidelines. The quality, purity, and concentrations of the RNA samples were assessed using a nano spectrophotometer (SPECTROstar Nano, BMG LABTECH, Ortenberg, Germany). Subsequently, cDNA synthesis was carried out utilizing the Takara PrimeScriptTM RT Reagent Kit with gDNA Eraser (Primerdesign, BLIRT S.A., Gdańsk, Poland). The procedures were conducted according to manufacturers’ instructions. Gene expression levels were determined via Real-Time PCR employing the SensiFast SYBR & Fluorescein Kit (Bioline, BIO-96020), following the manufacturer’s protocol. The specific forward and reverse primers for analyzed genes are referenced in **Table 2**. Real-Time PCR was performed with a CFX Connect™ Real-Time PCR Detection System (Bio-Rad) with the following cycling conditions: initial denaturation at 95°C for 2 minutes, followed by 40 cycles of denaturation at 95°C for 15 seconds, annealing for 15 seconds, and elongation at 72°C for 15 seconds. The relative expression levels of genes were normalized to the expression of glyceraldehyde 3-phosphate dehydrogenase (GAPDH) and calculated using the 2^-ΔΔCQ^ method.

**Table 2:** Sequences of primers used in qPCR.

Gene	Primer	Sequence 5’–3’	Amplicon length (bp)	Accession No.
*PINK*	F: R:	GCACAATGAGCCAGGAGCTA GGGGTATTCACGCGAAGGTA	298	XM_014737247.2
*Mfn1*	F: R:	AAGTGGCATTTTTCGGCAGG TCCATATGAAGGGCATGGGC	217	XM_023623336.1
*SIRT-1*	F: R:	ACCAACGGTTTTCATTCTTGTGA TTCGAGGATCTGTGCCAATCA	139	XM_023643979.1
*mTOR*	F: R:	GGGCAGCATTAGAGACGGTG ATGGTTGATTCGGTGTCGCA	221	XM_005607536.3
*BAX*	F: R:	CGAGTGGCAGCTGAGATGTT AAGGAAGTCCAGTGTCCAGC	153	XM_023650077.1
*BCL-2*	F: R:	TTCTTTGAGTTCGGTGGGGT GGGCCGTACAGTTCCACAA	164	XM_001490436.4
*BMP2*	F: R:	CGTCCTGAGCGAGTTCGAGT CGCCGGGTTGTTTTTCCACT	249	XM_023626137.1
*BMP4*	F: R:	TGGCTGTCAAGAATCATGGA TCCCCGTCTCAGGTATCAAA	149	XM_005605168.3
*BMP7*	F: R:	CATGCTGGACCTGTACAATGC TCCCCTTCTGGGATCTTGGA	255	NM_001195158.1
*OCL*	F: R:	CGCTACCTGGATCATTGGCT GAGCAGCTAGGGACGATGAG	257	XM_023641016.1
*ALP*	F: R:	AGGCCAAGATGAGGGTAGCA TCTCTGGCACTAAGGAGTTGGT	352	XM_014852743.1
*Runx2*	F: R:	ACTTTGCAGAGATGGGCCTC CTAGGAAGTCGGGATGGGGA	72	XM_023624254.1
*OPN*	F: R:	CATCGCCTATGCCCTTCCAG TGTGTGGTCATGGCTTTCGT	217	XM_001496152.4
*Coll1*	F: R:	GAAACTATCAATGGTGGTACCAAGT AGCAGCCATCTACAAGAACAGT	265	AB070839.1
*Parkin*	F: R:	CTGGAGGATTTAGTCCCGGAGC CCATGGCTGGAGTTGAACCTG	138	XM_005608126.3
*Beclin*	F: R:	GATGCGTTATGCCCAGATGC AACGGCAGCTCCTCTGAAAT	294	XM_001493225.4
*LAMP2*	F: R:	GCACCCCTGGGAAGTTCTTA ATCCAGCGAACACTCTTGGG	147	XM_014729146.2
*GAPDH*	F: R:	GATGCCCCAATGTTTGTGA AAGCAGGGATGATGTTCTGG	250	NM_001163856.1

PINK, PTEN Induced Kinase 1; Mfn1, Mitofusin 1; SIRT-1, Sirtuin 1; mTOR, Mechanistic Target of Rapamycin Kinase; BAX, Bcl-2–associated X; BCL-2, Apoptosis Regulator; BMP2, Bone Morphogenetic Protein 2; BMP4, Bone Morphogenetic Protein 4, BMP7, Bone Morphogenetic Protein 7; OCL, Osteocalcin; ALP, Alkaline Phosphatase, Biomineralization Associated; Runx2, RUNX Family Transcription Factor 2; OPN, Osteopontin; Coll1, Type I Collagen; Parkin, Parkine; Beclin, Beclin; LAMP2, Lysosomal-Associated Embranę protein; GAPDH, Glyceraldehyde-3-Phosphate Dehydrogenase.

### 2.11 Mitochondria isolation and staining

Mitochondria were isolated from osteogenic cells using the Mitochondria Isolation Kit for Cultured Cells (Thermo Scientific, Warsaw, Poland) according to the manufacturer’s protocol. Cells were suspended in 800 µl of reagent A containing proteinase inhibitor cocktail. The cells were then vortexed and kept on ice. Then 10 µl of Reagent B was added. The cells were vortexed again and incubated for 5 minutes on ice. After that, 800 µl of reagent C with proteinase inhibitor cocktail was added. The samples were centrifuged at 700g for 10 minutes at 4°C. The supernatant was transferred to a new Eppendorf tube and centrifuged at 12000g for 15 minutes at 4°C. The resulting mitochondrial pellet was washed in PBS, centrifuged again at 12000g for 15 minutes at 4°C and stained with MitoRed dye (1:1000) at 37°C for 30 min.-Stained mitochondria were afterwards washed with PBS, spread onto a microscope slide and captured under a Confocal laser scanning microscope (Observer Z1 Confocal Spinning Disc V.2 Zeiss). The intensity of mitochondrial staining was analyzed using the ‘Colour Pixel Counter’ plugin in Fiji, formerly known as ImageJ Software (version 1.52n, Wayne Rasband, National Institutes of Health, USA), with threshold values set at 49, 50, and 51.

### 2.12 Statistical analysis

The generated data were analyzed by one-way variance analysis (ANOVA) using GraphPad Prism Software 9 (San Diego, USA) and *post-hoc* Tukey’s test. Statistically significant results were marked with an asterisk, for: *p <0.05* (*), *p <0.01* (**), *p <0.001* (***) and *p < 0.0001* (****). Results are presented as statistical mean ± SD from at least three independent experiments with four technical repetitions.

## 3 Results

### 3.1 SHBG cytocompatibility assessment in undifferentiated equine ASCs CD34^+^ cells

**Figure 1:**
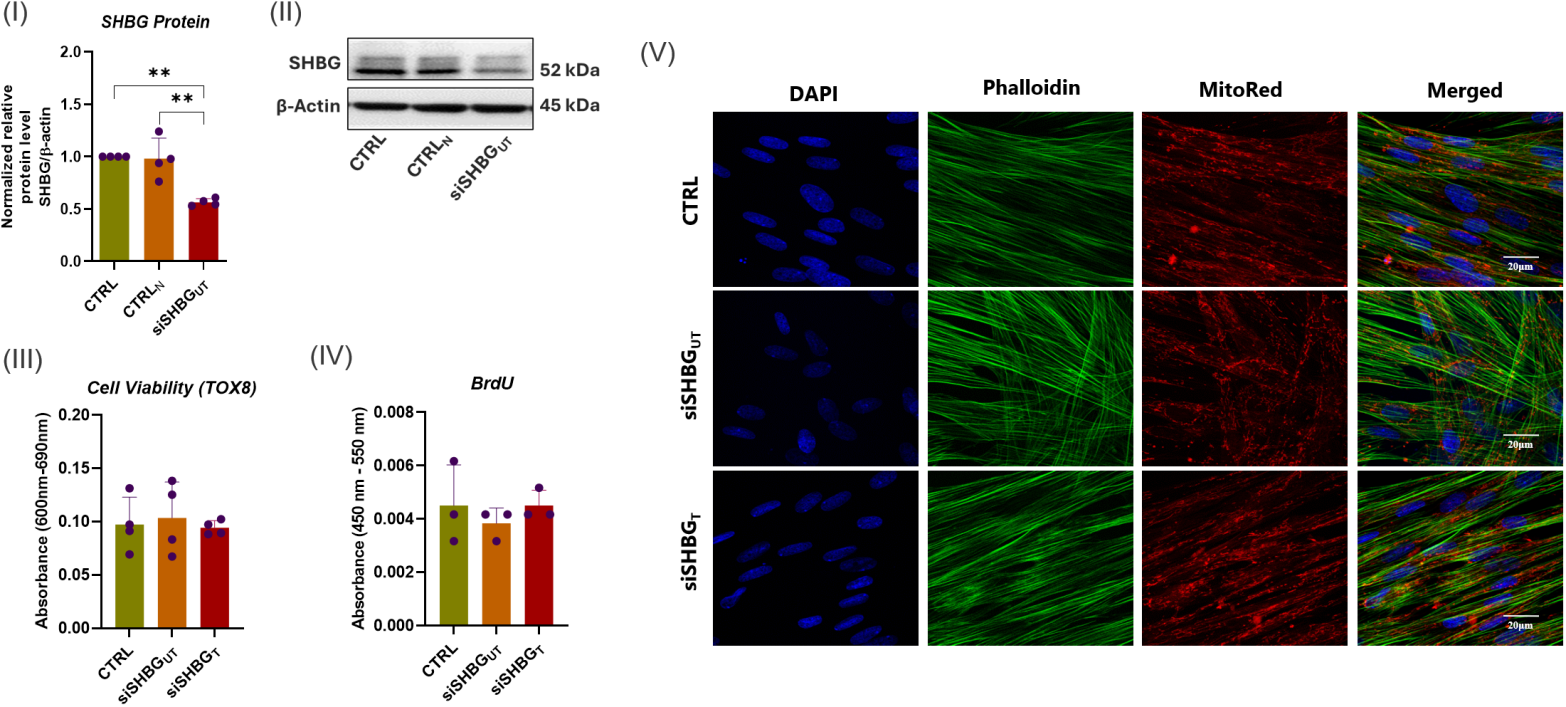
SHBG knockdown efficiency and cytocompatibility of SHBG protein with equine ASCs. (I) Quantitative analysis of SHBG protein expression relative to β-Actin and native control cells. (II) Representative immunoblots for SHBG and β-actin proteins. (III) Cell viability evaluated using the resazurin-based assay; (IV) Cell proliferation assessed through the incorporation of BrdU into neosynthesized DNA; (V) Cell morphology analyzed under a confocal microscope. Representative data are shown as mean ± SD. CTRL, ASCs untreated cells; CTRL_N_, ASCs cells treated with a scrambled siRNA as a negative control; siSHBG_UT_, ASCs cells with silenced SHBG; siSHBG_T_, ASCs cells with silenced SHBG and treated with 50 nM SHBG. **Statistical significance at p < 0.01.

In order to explore the role of SHBG in ASCs biology and its potential impact on their osteogenic potential, SHBG protein expression has been knocked down using targeted predesigned small interfering RNA (siRNA) approach, allowing to assess its functional implications. As shown in **Figure 1** I, the transfection of ASCs with SHBG siRNA targeting the exon 5 resulted in a significant reduction of SHBG relative protein level by approximately 56.46% compared to untransfected ASCs and siRNA negative control group (*p<0.01*), confirming the effectiveness of the siRNA in downregulating SHBG protein expression.

To assess the impact of SHBG on cellular dynamics and verify its cytocompatibility, alterations in morphology, viability and proliferation rate were examined following the suppression of SHBG and the introduction of exogenous SHBG. The deficiency of SHBG did not have a notable influence on cell viability and proliferation (**Figure 1** III and IV). Similarly, the addition of exogenous SHBG to compensate for the deficiency did not affect the aforementioned parameters. In terms of cell morphology, undifferentiated cells exhibited comparable geometry and size with native cells after SHBG suppression. The introduction of exogenous SHBG did not changed phenotype. The organization of the cytoskeleton, shape of nuclei, and their positioning did not show any noteworthy alterations (**Figure 1** V).

**Figure 2:**
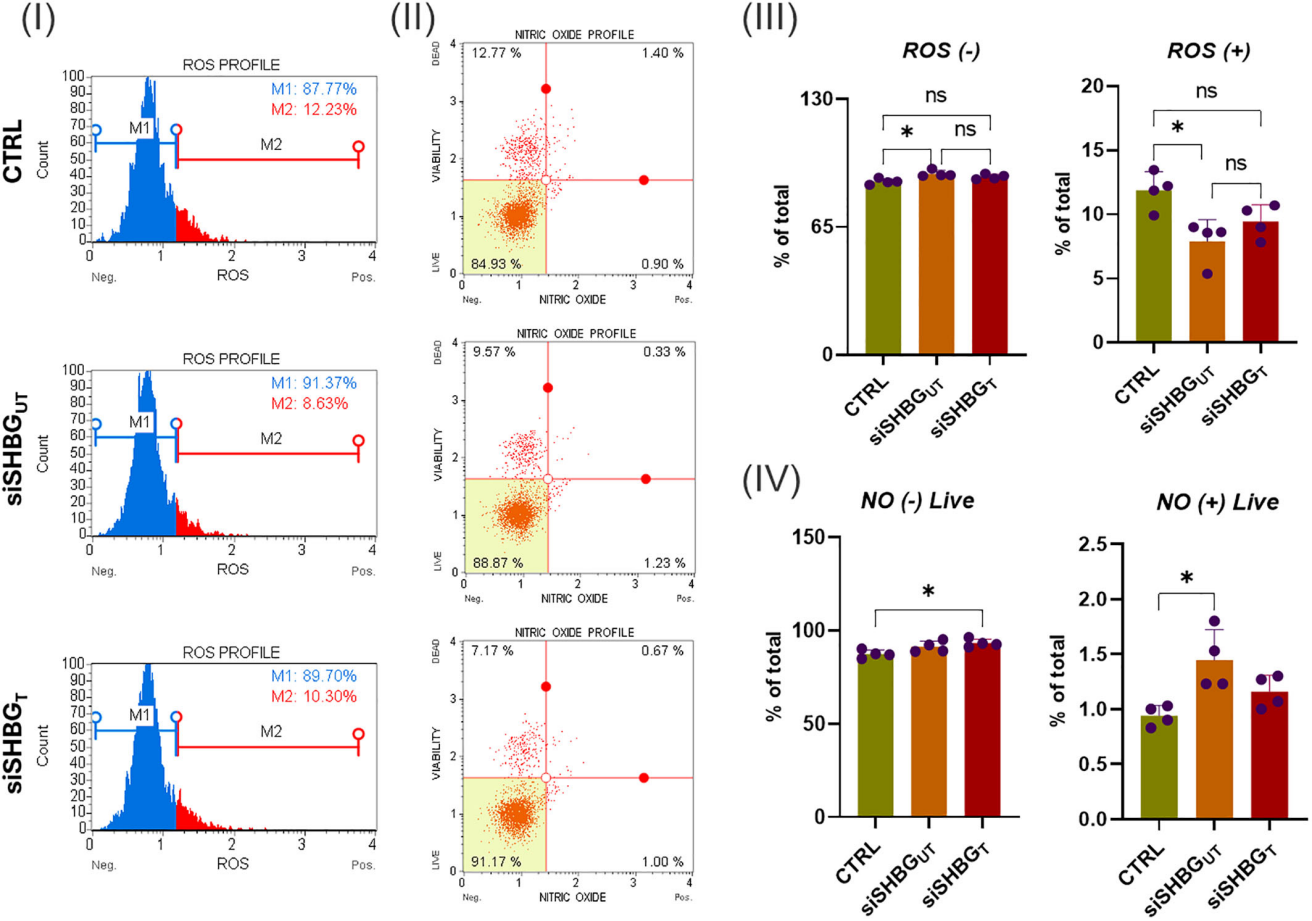
Oxidative stress status in equine ASCs following SHBG treatment. (I) Representative ROS assay plots, M1: negative cells, ROS^(–)^ and M2: cells with ROS activity, ROS^(+)^. (II) Representative nitric oxide (NO) measurement profiles. (III) Histograms depicting the percentage of cells negative and positive for ROS. (IV) Graphs showing NO content distribution in ASCs. Representative data are shown as mean ± SD. CTRL, ASCs untreated cells; siSHBG_UT_, ASCs cells with silenced SHBG; siSHBG_T_, ASCs cells with silenced SHBG and treated with 50 nM SHBG. *Statistical significance at p < 0.05.

The levels of oxidative stress were evaluated after suppressing SHBG by examining the accumulation of intracellular ROS using a Muse^®^ Oxidative Stress Kit (**Figure 2**). Interestingly, cells lacking SHBG showed a better response to ROS compared to the original and externally supplemented cells (**Figure 2** III). The depletion of SHBG led to an increased abundance of cells negative for ROS, as compared to the native cells. The addition of SHBG to silenced cell lines did not yield any changes. Additionally, the number of cells that were tested positive for ROS was downregulated in the SHBG-silenced cell lines when compared to the original cells. Treating the silenced cells with exogenous SHBG partially restored the cell population to its native state (**Figure 2** III). Surprisingly, the results confirmed a decrease in the levels of intracellular ROS in SHBG-depleted cells, while there was a noticeable increase in the ROS signal after applying exogenous SHBG protein. Furthermore, it has been established that SHBG plays a role in modulating the synthesis of nitric oxide (NO) (24). The results showed that knocking down SHBG did not affect the abundance of NO-negative cells but significantly increased the number of NO-positive cells compared to the native cells. Complementing the SHBG deficient group reversed the negative effect of nitric oxide on the cells (**Figure 2** IV).

### 3.2 Impact of SHBG exposure on the osteogenic potential of equine ASCs CD34^+^ cells

**Figure 3:**
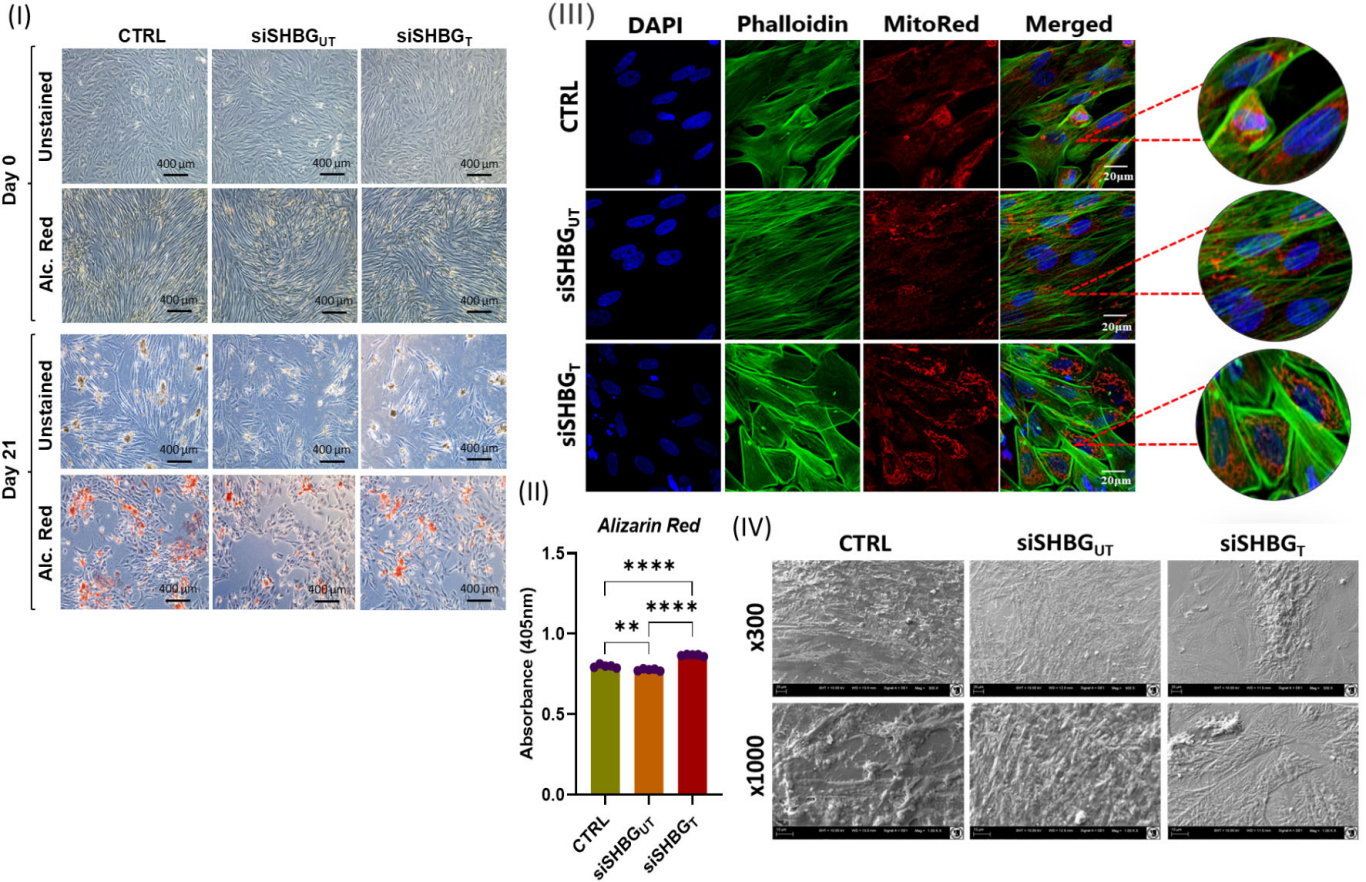
Morphological changes in Equine ASCs osteogenic cells under SHBG treatment. (I) Inverted light microscope micrographs of cells at day 0 and day 21 of osteogenesis unstained and Alizarin Red (Alc. Red) stained. (II) Quantitative measurement of alizarin red stain retained by specimens. (III) Confocal observation of osteogenic cells stained for nuclei with DAPI, cytoskeleton with Phalloidin and mitochondria with MitoRed. (IV) Cell surface and shape assessed by scanning electron microscopy (SEM). Representative data are shown as mean ± SD. CTRL, ASCs untreated cells; siSHBG_UT_, ASCs cells with silenced SHBG, siSHBG_T_, ASCs cells with silenced SHBG and treated with 50 nM SHBG. Statistical significance: p < 0.01 (**), p < 0.0001 (****).

Cell morphology was evaluated on the first day and after 21 days of osteogenic differentiation. Observations with inverted light microscope showed that after 21 days, SHBG deficient cells exhibit an unchanged fibroblast-like morphology, reflecting a relatively poor differentiation extend. The exposure to SHBG protein during the differentiation to SHBG deficient cells displayed marked change in cellular morphology, the appearance of multilayered cellular aggregates with cuboidal cells was noted reflecting osteogenic cells (**Figure 3** I and III).

In order to determine the osteogenic differentiation, the formation of extracellular mineralized matrix was measured by Alizarin Red staining. Significant decrease was observed for SHBG deficient cells in comparison to native cells (**Figure 3** II). Incubation of siSHBG cells with exogenous SHBG enhanced the mineralization of bone matrix compared with native and SHBG deficient cells, as evidenced by the substantial increase in the captured Alizarin Red staining within the matrix. Scanning electron microscopy confirmed the osteogenic differentiation in control and siSHBG cells treated with SHBG protein (**Figure 3** IV).

**Figure 4:**
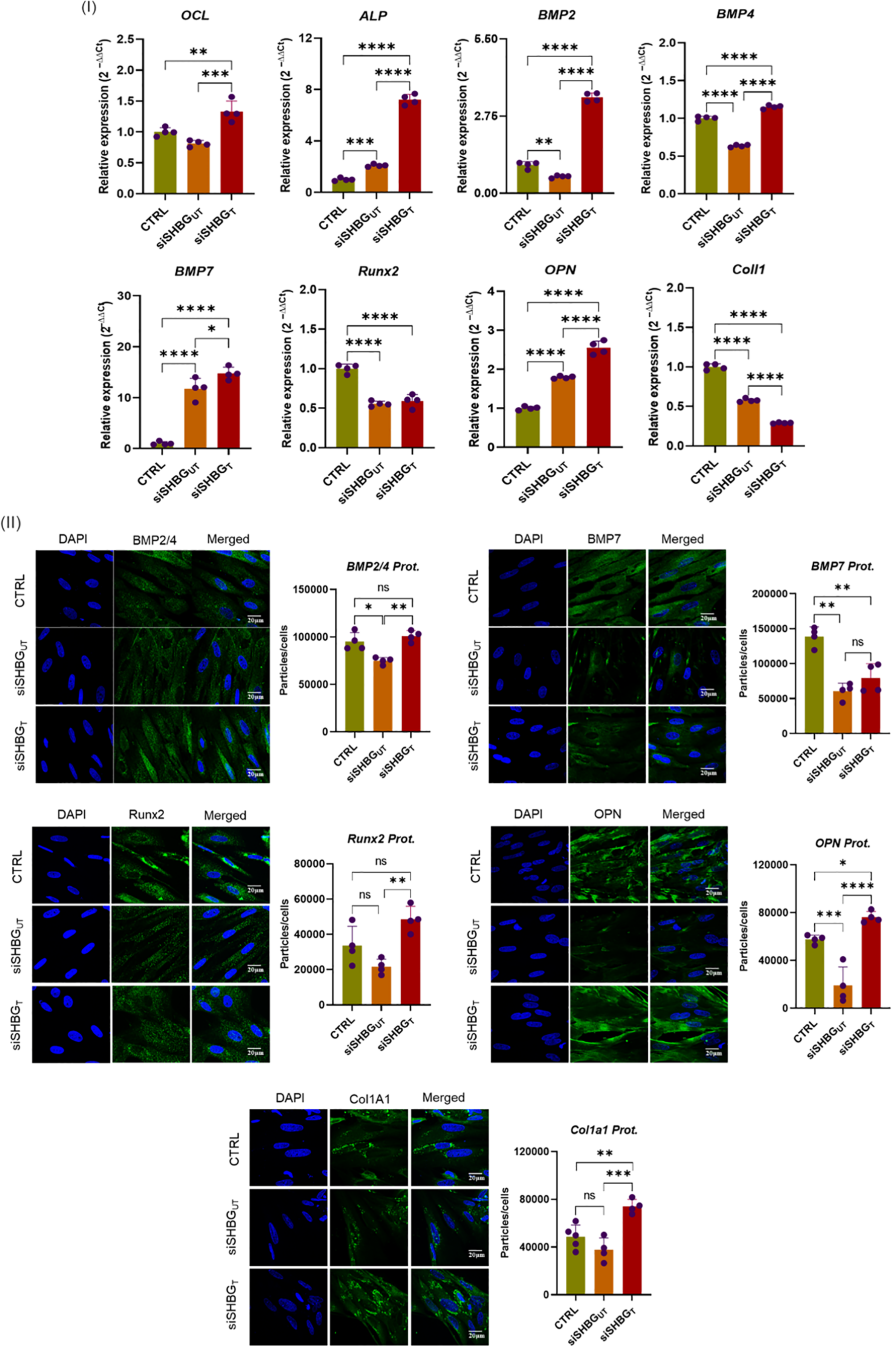
Differential expression of key osteoconductive mediators under SHBG influence in equine osteogenic ASCs. (I) Relative gene expression of master osteogenesis regulators; (II) Immunofluorescent staining and quantitative determination of pro-osteogenic proteins abundance. Representative data are shown as mean ± SD. CTRL, ASCs untreated cells; siSHBG_UT_, ASCs cells with silenced SHBG; siSHBG_T_, ASCs cells with silenced SHBG and treated with 50 nM SHBG. Statistical significance: p < 0.05 (*), p < 0.01 (**), p < 0.001 (***) and p < 0.0001 (****).

To clarify the influence of SHBG in molecular events leading to the formation of new bone, expression of osteogenic biochemical markers was investigated using RT-PCR and fluorescence immunohistochemistry along the course of ASCs differentiation. ALP and osteocalcin (OCL) are considered as initial markers of osteoblast differentiation. Thus, investigation of its transcript levels showed that the lack of SHBG lead to slight overexpression of ALP (*p<0.01*) while no changes were observed for OCL compared with native cells. External addition of SHBG to siSHBG_UT_ cells results in a significant increase of both ALP (*p<0.0001*) and OCL (*p<0.001*) compared with native and SHBG deficient cells (**Figure 4** I). Furthermore, the RT-PCR analysis of SHBG deficient cells showed a substantial downregulation of BMP2 (*p<0.01*), BMP4 (*p<0.0001*), RUNX2 (*p<0.0001*) and COL1 (*p<0.0001*) transcript levels, compared with native cells. Exogenous addition of SHBG markedly upregulated the expression of BMP2 (*p<0.0001*), BMP4 (*p<0.0001*), while decreasing COL1 (*p<0.0001*) and did not affect RUNX2 transcript level in comparison with siSHBG_UT_. Transcript level of BMP7 (*p<0.0001*) and OPN (*p<0.0001*) were found upregulated both in SHBG deficient and complemented cells with the excess of the latter, compared with native cells (**Figure 4** I). Interestingly, expression of osteogenesis regulators at proteins level did not completely correspond to transcript levels. Immunohistochemistry revealed the critical downregulation of all tested osteoconductive proteins in SHBG deficient cells in opposition to both native and SHBG treated cells (**Figure 4** II). Exposure to exogenous SHBG protein to SHBG deficient cells restored the protein levels of BMP2, BMP4, and RUNX2 to comparable levels with native cells proteins, while COL1A1 (*p<0.01*) and OPN (*p<0.05*) proteins were found at higher abundance in the SHBG treated cells compared to native control (**Figure 4** II).

### 3.3 Effect of SHBG treatment on ASCs cellular homeostasis during osteogenic differentiation

**Figure 5:**
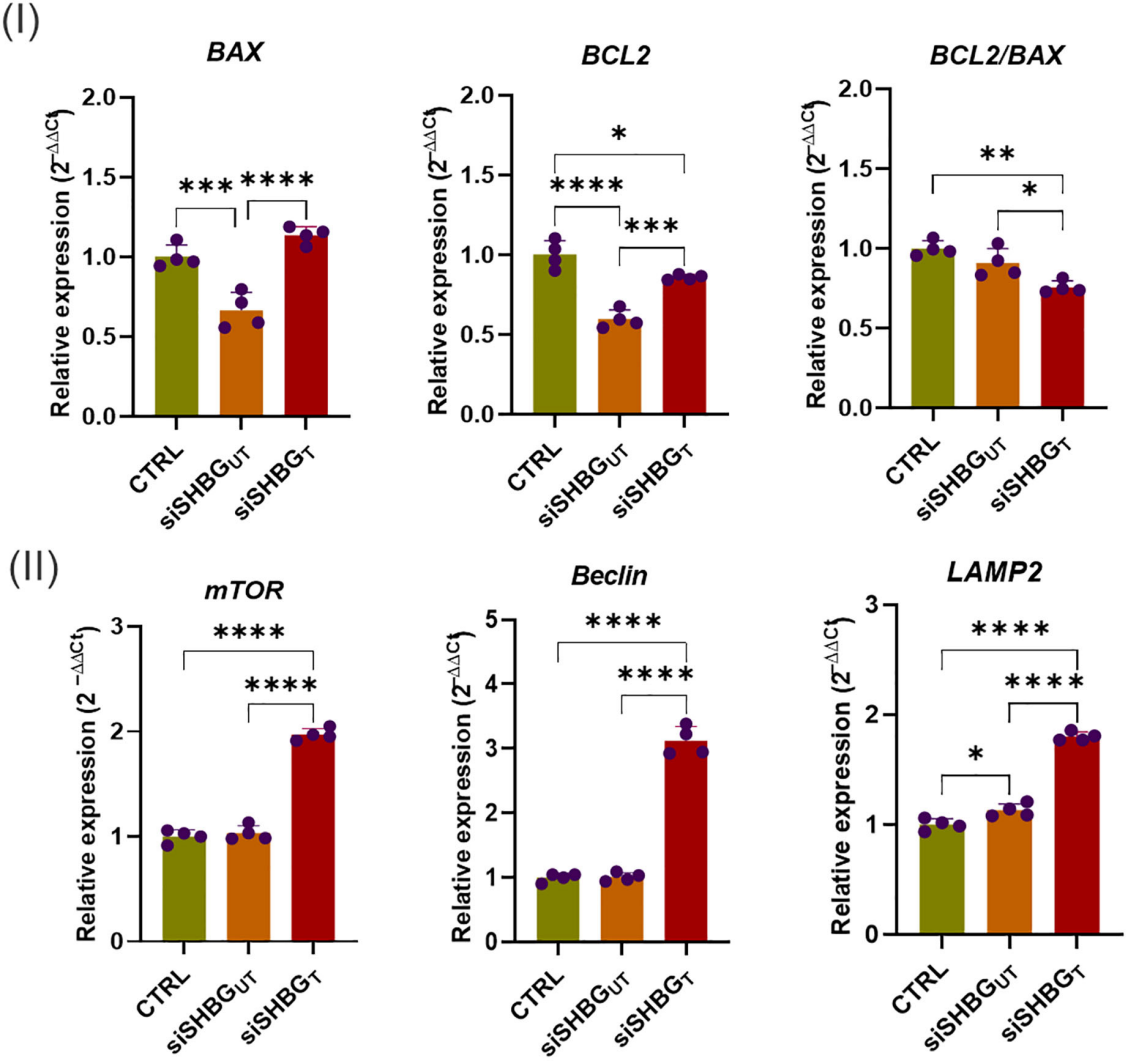
Gene transcription profiles of selected cellular integrity markers following SHBG application in equine osteogenic ASCs. (I) mRNA levels of key apoptosis regulators; (II) Transcripts abundance of autophagy-associated modulators. Representative data are shown as mean ± SD. CTRL, ASCs untreated cells; siSHBGUT, ASCs cells with silenced SHBG; siSHBGT, ASCs cells with silenced SHBG and treated with 50 nM SHBG. Statistical significance: p < 0.05 (*), p < 0.01 (**), p < 0.001 (***) and p < 0.0001 (****).

The importance of apoptosis in skeletal tissues and its functional relationship with bone turnover have been recently investigated by various authors to understand whether and, if so, how the mechanism of programmed cell death interferes with bone modelling (23, 25, 26). Here, expression of genes mediating apoptosis and autophagy during osteogenesis was investigated (**Figure 5**). mRNA expression of anti-apoptotic BCL2 (*p<0.0001*) and pro-apoptotic BAX (*p<0.001*) was significantly reduced in SHBG deficient cells in comparison to native cells. Application of exogenous SHBG triggered a remarkable increase of BAX (*p<0.0001*) and BCL2 (*p<0.001*) mRNAs (**Figure 5** I). By calculating BCL2 to BAX ratio expression, the susceptibility of cells to apoptosis was evaluated. Upon SHBG deficit, BCL2/BAX ratio was not significantly changed, however the treatment of cells with exogenous SHBG protein resulted in an important drop of the corresponding BCL2/BAX (*p<0.01*) ratio in comparison to native and silenced cells, suggesting a restoration of the apoptotic program upon SHBG application (**Figure 5** I). Autophagy markers mTOR, Beclin and LAMP2 were further determined at day 21 of osteogenesis (**Figure 5** II). While there was a significant upregulation of LAMP2 (*p<0.05*) mRNA and no changes in mTOR and Beclin transcripts in SHBG deficient cells, addition of 50 nM SHBG protein led to immense enhancement of mTOR (*p<0.0001*), Beclin (*p<0.0001*) and LAMP2 (*p<0.0001*) genes expression, compared with both native and silenced control cells.

### 3.4 Modulation of mitochondrial dynamics and morphogenesis in ASCs by SHBG during *in vitro* bone development

**Figure 6:**
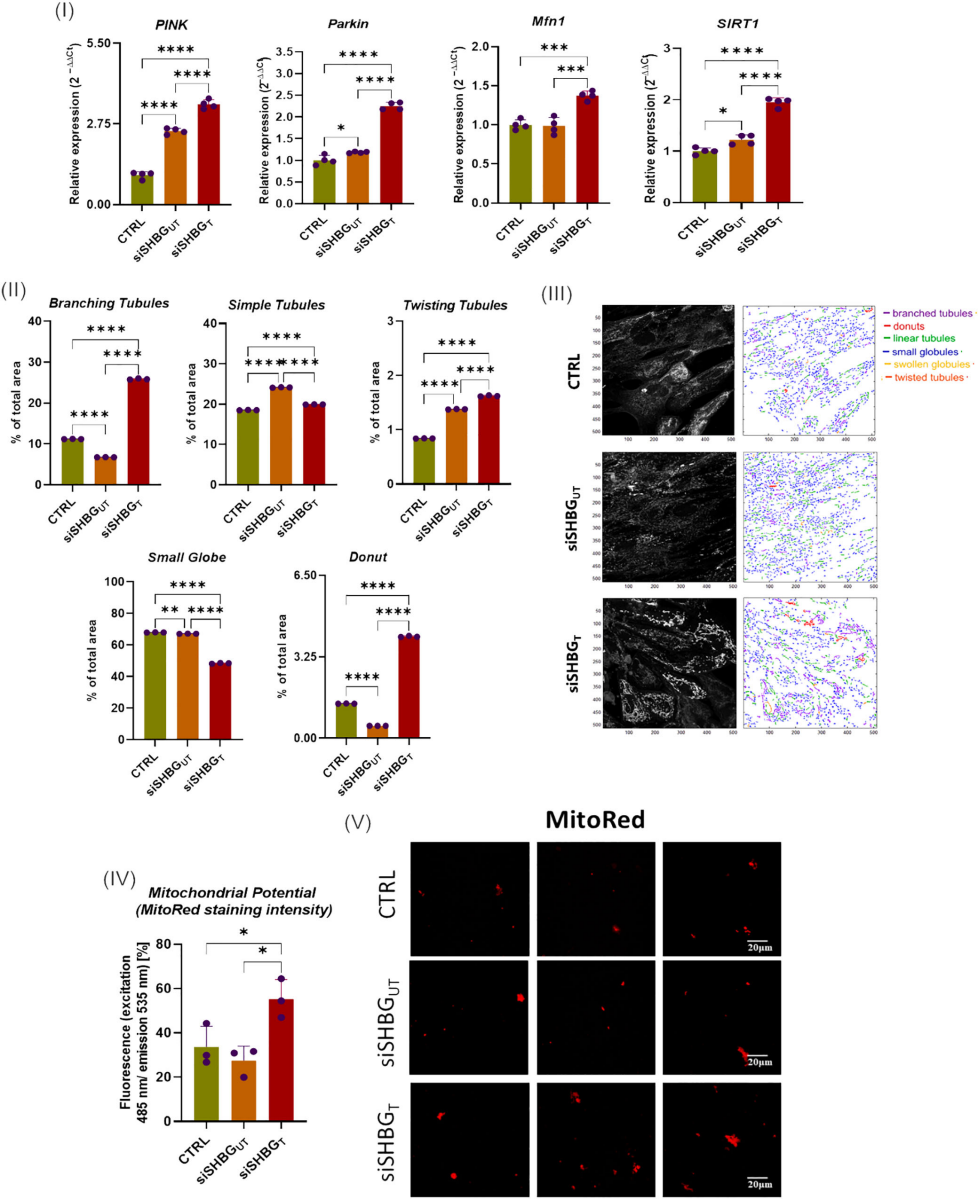
Mitochondrial dynamics status in equine ASCs during osteogenesis and SHBG treatment. (I) Relative gene expression of mitochondrial dynamics and mitophagy key regulators; (II) Mitochondrial subtypes distribution; (III) Mitochondrial Network morphology mapped with MicroP software; (IV) mitochondrial transmembrane potential determined as MitoRed sequestration percentage; (V) Confocal photomicrographs illustrating the mitochondrial network stained with fluorescent Red MitoTracker. Representative data are shown as mean ± SD. CTRL, ASCs untreated cells; siSHBGUT, ASCs cells with silenced SHBG; siSHBGT, ASCs cells with silenced SHBG and treated with 50 nM SHBG. Statistical significance: p < 0.05 (*), p < 0.01 (**), p < 0.001 (***) and p < 0.0001 (****).

Assessment of mitochondrial biogenesis, dynamics and mechanistic covers mRNA measurement of PINK, PARKIN, MFN1 and SIRT1 mRNAs. The obtained quantitative RT-qPCR data indicated that SHBG silencing resulted in a significant upregulation of PINK (*p<0.0001*), PARKIN (*p<0.05*) and SIRT1 (*p<0.01*) mRNAs, while abundance of MFN1 mRNA did not change compared to native cells (**Figure 6** I). In cells treated with exogenous SHBG, upregulation of PINK (*p<0.0001*), PARKIN (*p<0.0001*) MFN1 (*p<0.001*) and SIRT1 (*p<0.0001*) was observed in comparison to SHBG silenced cells.

In order to verify whether SHBG metabolic effects are associated to the cellular mitochondrion, various mitochondrial health-related parameters have been evaluated. Mitochondrial morphology in native, SHBG-silenced, and SHBG-treated silenced cells was analyzed using the MicroP software (**Figure 6** II and III). The obtained quantitative data showed that SHBG silencing engendered a switch from branched interconnected mitochondria to simple and twisted tubular structure (**Figure 6** II). Similarly, a lower proportion of donut-like mitochondria was found in the same group of cells (*p<0.0001*). Addition of exogenous SHBG to silenced cells results in an increased abundance of branching, donut and twisting mitochondria contrasted by a decrease in small globes and simple tubules in comparison to silenced and native cells.

In addition, the mitochondrial membrane potential was measured using mitoRed staining (**Figure 6** IV and V). Surprisingly, mitochondria of silenced cells did not display any significant changes in the mitochondrial potential, while mitochondria of SHBG deficient cells complemented with exogenous SHBG protein exhibited higher transmembrane potential compared to both silenced and native cells (*p < 0,05*).

## 4 Discussion

Musculoskeletal injuries and chronic degenerative diseases are significant concerns in horses, affecting their health, performance, and overall well-being. These conditions encompass a wide range of affections and structural abnormalities including among others lameness, osteoarthritis, tendon and ligament injuries and bone disorders. The status of musculoskeletal diseases in horses can vary depending on factors such as age, breed, management practices, and the demands placed on the animals. The subsequent healing processes often result in incomplete functional regeneration of the affected tissues, which contributes to elevated rates of reinjury, progression of tissue degeneration, and the onset of chronic morbidity (27).

Sex Hormone-Binding Globulin (SHBG) is a glycoprotein primarily known for its role in binding and transporting sex hormones, such as testosterone and estradiol, in the bloodstream. While the direct effects of SHBG on bone metabolism are not fully elucidated, it is believed to indirectly influence bone health through its interactions with sex hormones and other metabolic pathways (1). Hence, in the present study, we showed that SHBG enhances the expression of key modulators involved in osteogenic differentiation of equine ASCs, highlighting a potential use of SHBG for bone regeneration.

The osteogenic potential of ASCs is closely linked to their ability to undergo lineage-specific differentiation towards the osteoblastic phenotype. Defective ASCs have shown dysregulated expression of key osteogenic markers, such as alkaline phosphatase (ALP), osteocalcin (OCL), and collagen type I (ColI), which are essential for bone formation and mineralization. This dysregulation can disrupt the normal process of osteogenesis and result in the formation of immature or dysfunctional bone tissue (28). In this investigation, we found that incubation of ASCs with SHBG protein during osteogenesis induction resulted in an increased expression of osteogenesis master regulators including BMPs, OCL, ALP, Col1A and OPN.

Early osteogenesis is governed by the activation of the BMPs/Runx2 arm, which is considered as a central initiator of MSCs commitment along an osteoblastic lineage. A number of BMPs isotypes are thus involved in the osteogenic differentiation of MSCs. Particularly, BMP-2, BMP-4 and BMP-7 have been identified as paramount osteoconductive proteins regulating the osteogenic potential of ASCs (29). BMP-2 activation and/or stimulation triggers the rapid expression of osteocalcin, which irreversibly culminates to bone matrix formation (30). Likewise, BMP-4 acts downstream the Smad pathway that stimulates expression of osteogenic transcription factors, including Runx2, which orchestrate the differentiation of ASCs into osteoblasts. Moreover, BMP-4 promotes the synthesis and deposition of bone matrix components by osteoblasts, through the upregulation of genes encoding proteins such as collagen type I, osteopontin, and osteocalcin (31, 32). Our data demonstrated that SHBG treatment increases the expression of BMP-2 and BMP-4 and subsequent targets OCL, OPN and ALP, suggesting that SHBG might promote early osteogenesis stages in equine ASCs, by targeting BMPs family proteins. Similarly, an increased expression of BMP-7 markers correlated with an overexpression of ALP following SHBG application, confirms the probable implication of SHBG in the bone matrix mineralization process, as BMP-7 protein has been largely recognized as a key modulator of calcium mineralization and bone remodeling (33). One of the most likely mechanisms by which SHBG impacts the expression of BMP family members lies in the activation of the cAMP/PKA pathway. Indeed, previous findings strongly suggest that SHBG binding to its megalin receptor and/or its internalization induces a significant elevation in cytosolic levels of the cyclic monophosphate nucleotides (cAMP) second messenger (34–36). Increased cAMP is known to directly activate protein kinase A (PKA), which subsequently phosphorylates the cAMP-response element-binding protein (CREB). Phosphorylated CREB then binds to the cAMP-response element (CRE) in the promoter sequences of target genes, including those in the BMP family such as BMP-2 and BMP-7, thereby enhancing their transcription and expression (37). Ultimately, this suggests that the cAMP-PKA/CREB/CRE signaling pathway might be a crucial mechanism by which SHBG enhances BMP expression, thereby promoting osteogenic process and effective bone matrix mineralization. However further in-depth experiments are required to verify this hypothesis and highlight the exact mechanisms underlying the osteogenesis-inducing properties of SHBG.

Apoptosis plays an important role during MSCs osteogenic commitment, and influences the balance between proliferation and differentiation of osteoprogenitor cells. Programmed cell death acts as a regulatory mechanism that controls the proportion of precursor cells available for differentiation into osteoblasts, thereby contributing to bone formation (38). Accordingly, apoptosis of osteoblasts and osteocytes is essential for the proper matrix mineralization and bone turnover (39). During bone matrix mineralization, osteoblasts become embedded within the mineralized matrix and differentiate into osteocytes. Some of these osteocytes undergo apoptosis, creating small lacunae within the bone matrix. This apoptotic process facilitates the deposition and organization of hydroxyapatite crystals, contributing to the mechanical strength and integrity of the bone (40). Obtained data showed that SHBG depletion resulted in a loss in basal apoptotic capacity, evidenced by a decrease in the expression of Bax pro-apoptotic factor. Furthermore, the ASCs cells treated with exogenous SHBG displayed a restored expression of Bax and a corresponding decreased Bcl2/Bax ratio, indicating that SHBG might maintain adequate apoptosis during bone regeneration. These observations corroborate with our previous findings showing the SHBG interplay in mediating basal apoptotic fluxes (41).

During osteogenesis and bone tissue turnover, cellular homeostasis is maintained via autophagy, a highly conserved clearance machinery. Upon osteoblast differentiation initiation, autophagy is upregulated, while its inhibition has been shown to impair both osteoblast differentiation and mineralization. Autophagy promotes the degradation of damaged organelles and facilitates the clearance of cytoplasmic contents to provide energy and building blocks necessary for osteoblast differentiation and matrix synthesis (42, 43). In this study, SHBG protein has been found to enhance the expression of autophagy master regulators including Lamp2, mTOR and Beclin. Proper differentiation of MSCs into osteoblasts encompasses two distinct phases of autophagic signaling pathways. Initially, an early event characterized by the induction of the AMP-activated protein kinase (AMPK)/mTOR signaling axis, and a subsequent later stage marked by the activation of the Akt/mTOR signaling arm (44). The engagement of BMP2 with its receptor further prompts the expression of specific autophagy-related proteins, such as beclin 1 and Lamp2, thereby priming the autophagy machinery. The resulting autophagosomes are successively utilized to cargo mineral crystals to the extracellular matrix to fuel the bone mineralization process (45). Hence, SHBG emerges as a potential protein capable of promoting autophagy, thereby bolstering osteogenesis in ASCs and could represent a promising mechanism contributing to bone formation.

Mitochondria are integral organelles in osteogenesis, contributing to energy production, metabolic regulation, calcium homeostasis, ROS signaling, and apoptosis regulation during osteogenic differentiation and bone neoformation (46). To sustain cellular demands, mitochondria dynamically alter their morphology and activity via redox balancing, complemented by metabolic and quality control mechanisms (47). In this study, the application of SHBG protein during *in vitro* induced osteogenesis resulted in a visible reorganization of the mitochondrial network in equine ASCs. Treated cells exhibited increased proportion of branched and donut-shaped mitochondria compared to both SHBG-silenced and control cells. These differences in the mitochondrial morphology distribution evokes an enhanced adaptation of SHBG-treated cells to the osteogenic milieu. Indeed, Shum et al. (48) reported in their study a typical rearrangement of the mitochondrial network of osteogenic MSCs from an individualized mitochondrion in undifferentiated cells to an elongated and highly branched network after differentiation. These findings are in line with the present results showing a predominance of branched mitochondria as a result of SHBG treatment. This tendency further correlated with an increased expression of mitofusin 1 (MFN1), which is a key mitochondrial transmembrane GTPase that mediate the tethering and fusion of adjacent mitochondria (49).

Conversely, Sih and colleagues (50), demonstrated that formation of donut-like mitochondria substantially accelerates the osteogenesis of osteoprogenitor cells and the subsequent generation of mitochondrial-derived vesicles (MDVs) for extracellular secretion which is physiologically implicated in osteoblasts maturation. Similarly, the outcomes of our experiment demonstrated a higher abundance and co-existence of donut mitochondria in the SHBG treated group, which can be correlated to the observed increased Bax expression, as previous Bax deficiency was found to impair complete maturation of osteoblasts and has been associated to the prominent role of Bax in the fragmentation of mitochondria independently from apoptosis (51).

Mitochondrial dynamic mechanisms coordinate the balance of mitochondrial fusion, fission, and mitophagy, which collectively influence mitochondrial quality and function throughout the osteogenic differentiation process (46). The selective elimination and degradation of defective mitochondria though mitophagy has been largely implicated in the regulation pathways regulating osteoprogenitor cells fate, and was shown to be mostly mediated through the Pten-induced putative kinase 1 (PINK1)/Parkinson disease-related gene (Parkin) pathway (52). In this research, the exposure of ASCs to SHBG protein throughout the 21 days differentiation engendered a remarkable elevation in the mRNA level of both mitophagy mediators i.e. PINK1 and PARKIN, implying the promotion of mitophagy. The importance of PINK1 and PARKIN enzymes in maintaining osteogenic cells functions and differentiation potential has been previously documented. Lee and collaborators (53), indicated that PINK1 kinase expression was naturally increased during osteogenesis and its deficiency caused a critical suppression of osteogenic markers expression including ALP, bone sialoprotein (BSP), OCN, and OPN. Accordingly, other investigation demonstrated that Parkin overexpression accelerates osteoblastic differentiation of Bone marrow-derived MSCs by enhancing the expression levels of ALP, Runx2, and Col, while its deletion was found to critically refrain osteogenesis, showcasing the paramount role of PARKIN in sustaining the osteogenic potential of MSCs (54). Hence, the observed upregulation of PINK1 and PARKIN in response to SHBG treatment suggests that SHBG may enhance osteogenesis through its impact on mitochondrial dynamics and mitophagy. This aligns with the previously established role of SHBG in inhibiting Extracellular signal-regulated kinases 1 and 2 (ERK1/2) phosphorylation (55–57), which has been linked to improved mitochondrial function and the promotion of mitophagy, mainly through SGK1 phosphorylation and mTOR2 activation, leading to osteogenic differentiation and higher expression of key osteogenic factors to promote bone formation (58, 59). Taken together, these data strongly evidence the positive impact of SHBG on the osteogenic potential of equine ASCs, which might be primarily facilitated through its direct modulation of mitochondrial dynamics and the promotion of mitophagy.

The evidence of mitochondrial dysfunction playing a pivotal role in the pathogenesis of various metabolic diseases and aging has been primarily associated to their implication in modulating the redox balance and oxidative stress. Mitochondria generate free radicals, including nitric oxide (•NO), and are especially vulnerable to oxidative damage. Nitric oxide and reactive nitrogen species (RNS) in particular exert complex and diverse effects on mitochondrial function, leading to considerable damage of mitochondrial components such as proteins, lipids, and mtDNA. Such deterioration further disrupts mitochondrial membrane potential, triggering the release of pro-apoptotic factors and intensifying cell death pathways (60).

Our results showed that SHBG silencing led to a significant increase in NO+ cells, whereas the addition of exogenous SHBG reduced NO+ levels, although this reduction was not statistically significant. Despite the lack of previous studies establishing a direct link between the physiological effects of SHBG and fluctuations in NO levels, recent research on transgenic MC3T3-E1 preosteoblasts has shown that those expressing high levels of the HNF4α transcription factor exhibited increased osteogenic differentiation capacity, along with reduced NO levels and lower expression of nitric oxide synthases (NOS). In contrast, HNF4α-deficient cells displayed an opposing pattern (61). Given that HNF4α is known as a major determinant for SHBG expression (62), it is plausible that SHBG modulates NO levels during osteogenesis through HNF4α signaling. This connection indicates a potential regulatory pathway where HNF4α affects SHBG expression, which in turn influences nitric oxide levels and osteogenic differentiation. Further investigation is required to elucidate this interaction and its implications for bone formation.

## 5 Conclusion

The present study provides compelling evidence for the positive impact of SHBG on the osteogenic potential of equine ASCs. The treatment of ASCs with SHBG promoted the expression of key osteoconductive factors including BMP-2/4, ALP, OCL and OPN, enhanced the Lamp2/Beclin/mTOR autophagy axis and modulated mitochondrial dynamics. These findings offer new insights into SHBG’s role in bone formation and suggest its therapeutic potential for bone regeneration in equine medicine.

## Data Availability

The original contributions presented in the study are included in the article/supplementary material. Further inquiries can be directed to the corresponding author/s.
